# Supplementary material for: A drug-free cardiovascular stent functionalized with tailored collagen supports in-situ healing of vascular tissues
Source: Nat Commun. 2024 Jan 25;15:735. doi: 10.1038/s41467-024-44902-2 (PMC10810808; doi:10.1038/s41467-024-44902-2)
Supplement: Supplementary file 1 — Supplementary Information [file 41467_2024_44902_MOESM1_ESM.pdf]

A Drug-free Cardiovascular Stent Functionalized with Tailored Collagen Supports in-situ Healing of Vascular Tissues

Haoshuang Wu [a, #], Li Yang [a, #], Rifang Luo [a], Li Li [b], Tiantian Zheng [a], Kaiyang Huang [a], Yumei Qin [a], Xia Yang [c], Xingdong Zhang [a], Yunbing Wang*[, a,d]

[a] National Engineering Research Center for Biomaterials and College of Biomedical Engineering, Sichuan University, Chengdu 610065, China

[b] Institute of Clinical Pathology, West China Hospital of Sichuan University, Chengdu 610041, China

[c] Shanxi Key Laboratory of Functional Proteins, Shanxi Jinbo Bio-Pharmaceutical Co., Ltd., Taiyuan 030032, Shanxi, China

[d] Tianfu Jincheng Laboratory (Frontier Medical Center), Chengdu 610213, China

[#]: These authors contributed equally

*: Corresponding author

E-mail address: yunbing.wang@scu.edu.cn (Y. Wang).

This file contains:

- Supplementary Fig. 1-18
- Supplementary Table 1-5

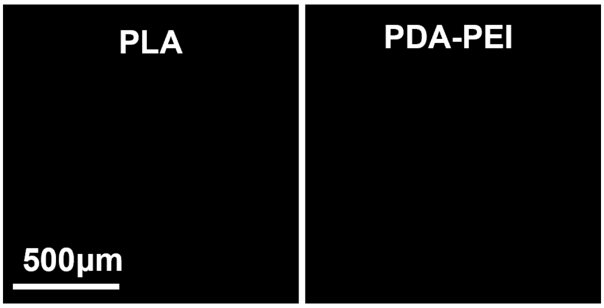

Supplementary Fig. 1 | Representative FITC fluorescence signals of bare, and PDA-PEI-coated PLA sheets. No fluorescence signals were observed in the control bare PLA and PDA-PEI groups. Scale bars, 500 μm. Five samples were repeated independently with similar results.

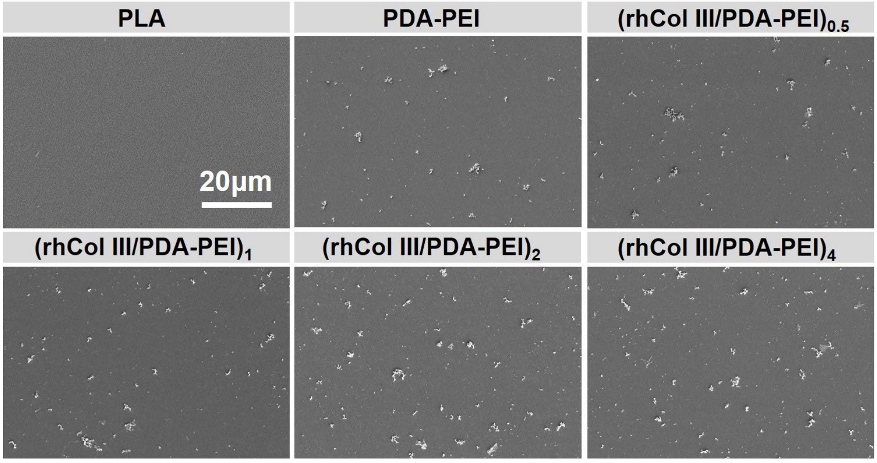

Supplementary Fig. 2 | Representative scanning electron microscopy (SEM) images of bare and (rhCol III /PDA-PEI)$_n$-coated Poly (l-lactic acid) (PLA) sheets (n=0.5, 1, 2, and 4). The PLA sheets were relatively smooth, while a gradually increased number of particles on (rhCol III/PDA-PEI)$_n$-coated PLA sheets were observed as more uniform and continuous as the feeding concentration of rhCol III increased. Scale bars, 20 μm. Five samples were repeated independently with similar results.

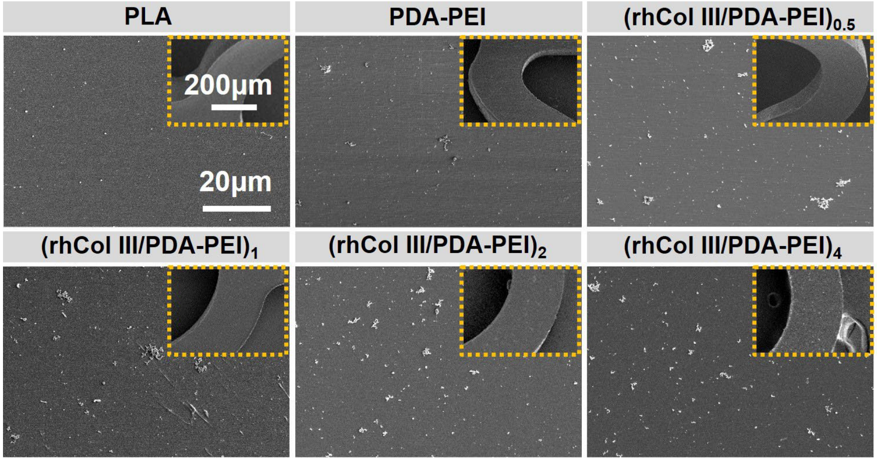

Supplementary Fig. 3 | Representative SEM images of the bare and (rhCol III /PDA-PEI)$_n$-coated PLA stents (n=0.5, 1, 2, and 4). The surface of PLA stents was relatively smooth, while the number of particles on the (rhCol III/PDA-PEI)$_n$-coated PLA stents gradually increased with increasing rhCol III feed concentration with more uniformity and continuity. Scale bars, 20 μm and 200 μm. Five samples were repeated independently with similar results.

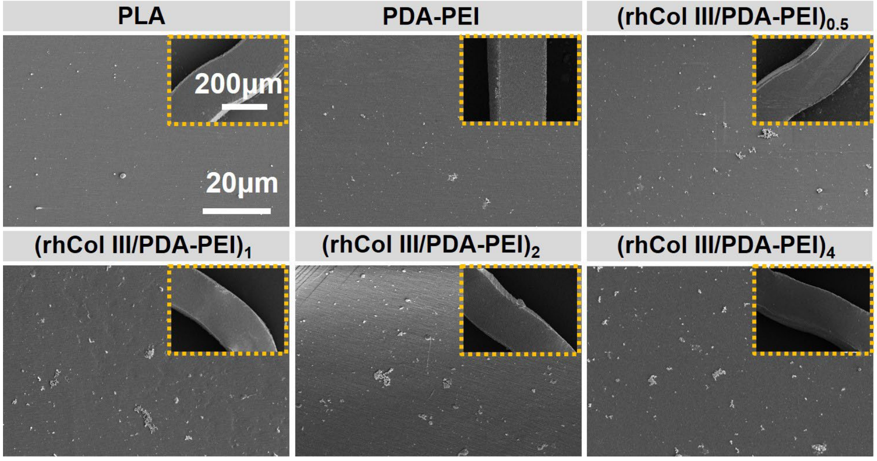

Supplementary Fig. 4 | Mechanical stability testing of the (rhCol III/PDA-PEI)$_n$ after balloon dilation in PBS at 37°C. Representative SEM images showing that the (rhCol III /PDA-PEI)$_n$ coatings remained connected without cracks. Scale bars, 20 μm and 200 μm. Five samples were repeated independently with similar results.

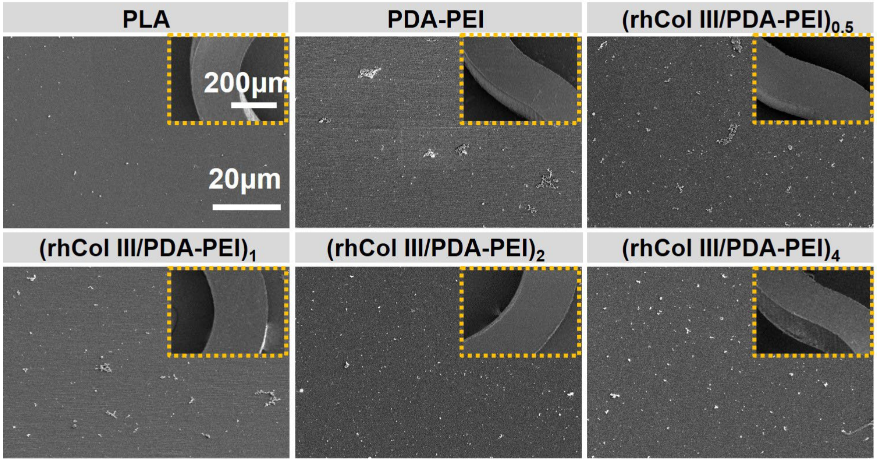

Supplementary Fig. 5 | Long-term stability of (rhCol III /PDA-PEI)$_n$ (n=0.5, 1, 2, and 4) coatings after 4 weeks of circulation under flowing system with bovine blood serum at 37°C. Representative SEM images showing that the strut surfaces of the dilated stents coated with (rhCol III /PDA-PEI)$_n$ (n=0.5, 1, 2, and 4) coatings. Scale bars, 20 μm and 200 μm. Five samples were repeated independently with similar results.

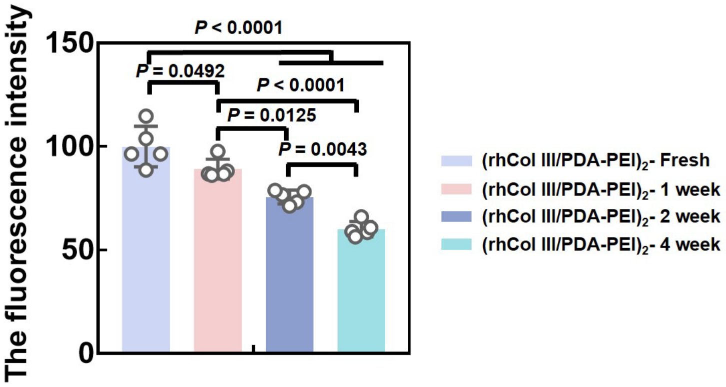

Supplementary Fig. 6 | Quantification of fluorescence intensity after balloon dilation in PBS at 37°. The fluorescence intensity of fresh (rhCol III/PDA-PEI)$_2$ coating was set as 100%, calculated by Image J software (n=5 independent samples). One-way ANOVA with Tukey's multiple comparisons was used for the comparisons. The data are presented as the mean ± SD (*P* values <0.05 were considered statistically significant).

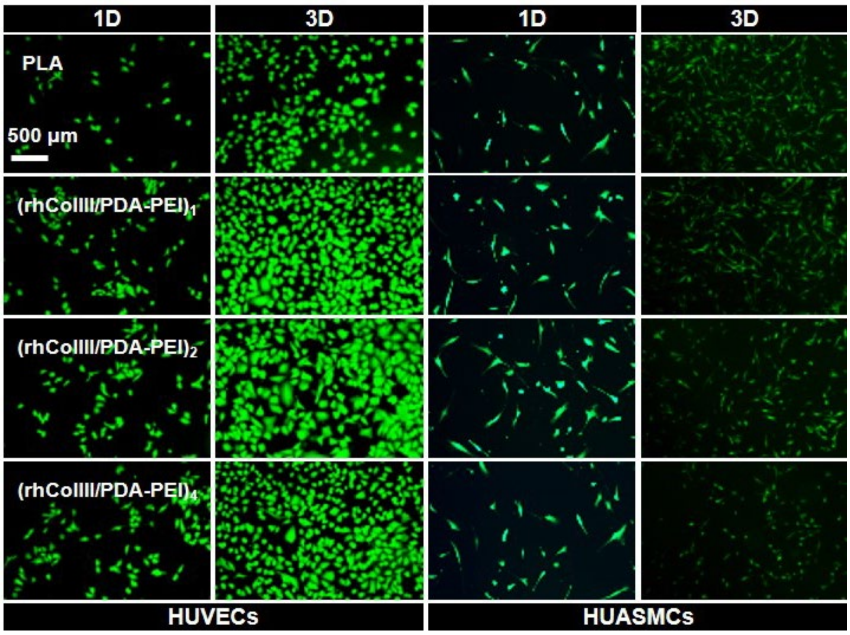

Supplementary Fig. 7 | Representative fluorescein diacetate (FDA) fluorescent staining (green) of the HUVECs and HUASMCs on uncoated- and (rhCol III /PDA-PEI)$_n$ coated-PLA sheets after incubation for 1 day and 3 days at 37 °C. FDA fluorescent images showing that (rhCol III/PDA-PEI)$_n$ coatings (n=0.5, 1, 2, and 4) favored the HUVECs adhesion but suppressed HUASMCs adhesion. Scale bars, 500 μm.

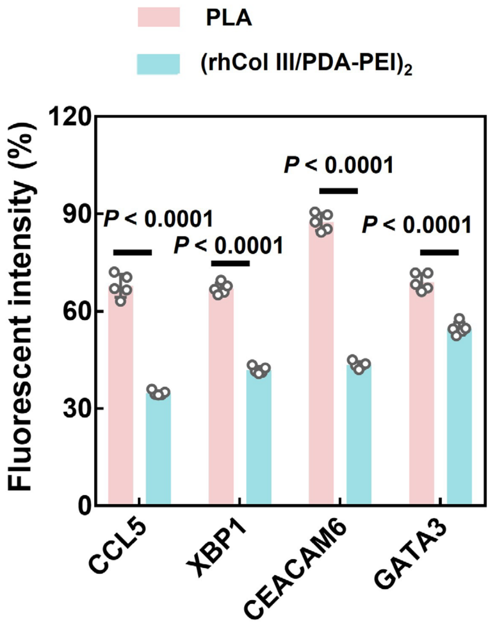

Supplementary Fig. 8 | Quantification of relative *CCL5*, *GATA3*, *XBP1*, and *CEACAM6* expression from corresponding immunofluorescence images. The (rhCol III/PDA-PEI)2 group down-regulated the above four genes compared to the PLA group (n=5 independent samples). Two-way ANOVA with Tukey's multiple comparisons was used for the comparisons. The data are presented as the mean ± SD (*P* values <0.05 were considered statistically significant).

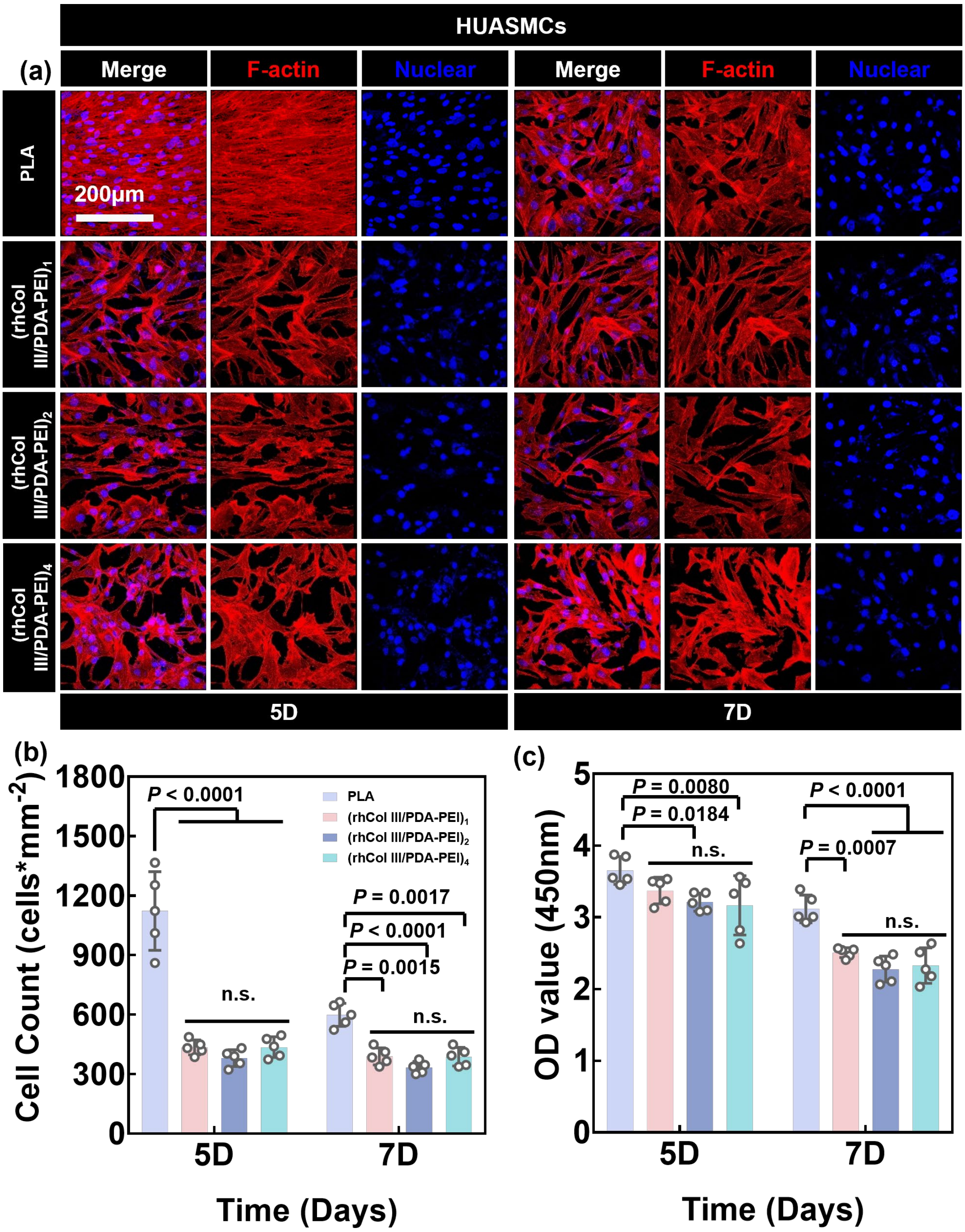

Supplementary Fig. 9 | (rhCol III/PDA-PEI)n (n=1, 2, and 4) inhibited the adhesion

and proliferation of HUASMCs via mediating the phenotypic switch. (a) Representative fluorescence images of rhodamine- (red) and DAPI- (blue) stained HUASMCs showing the (rhCol III/PDA-PEI)$_n$ coatings loaded with different amounts of rhCol III all suppressed HUASMCs compared with control PLA group. Scale bars, 200 μm. (b) Quantification of cell number and (c) Cell viability of HUVSMCs cultured on uncoated- and (rhCol III/PDA-PEI)$_n$-coated PLA sheets after 5 and 7 days of culture (n=5 independent samples). Two-way ANOVA was used for the comparisons in (b) and (c). The data are presented as the mean ± SD (*P* values <0.05 were considered statistically significant, and n.s. indicated not significant).

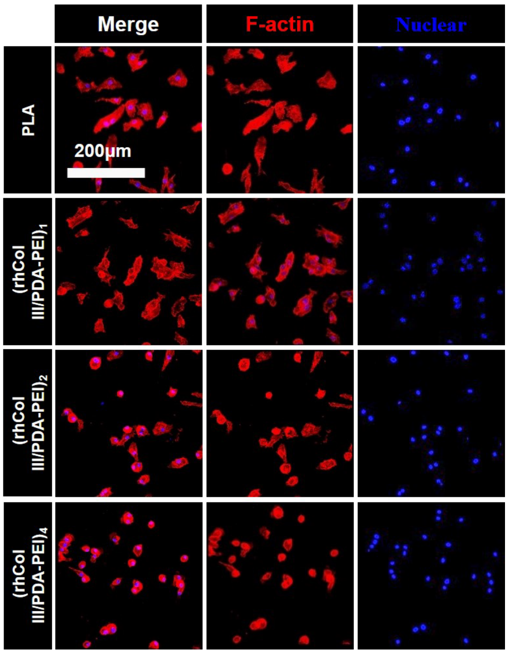

Supplementary Fig. 10 | (rhCol III/PDA-PEI)$_n$ coatings (n=1, 2, and 4) favor the HUVECs adhesion. Representative fluorescence images of rhodamine- (red) and DAPI- (blue) stained MBMMCs showing the control PLA group favored the elongating and stretching of MBMMCs compared with (rhCol III/PDA-PEI)$_n$ coatings (n=1, 2, and 4). Scale bars, 200 μm.

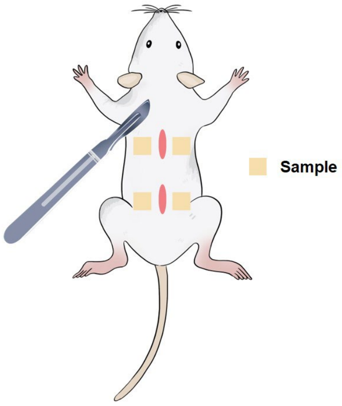

Supplementary Fig. 11 | Schematic diagram of the SD rat subcutaneous implantation model.

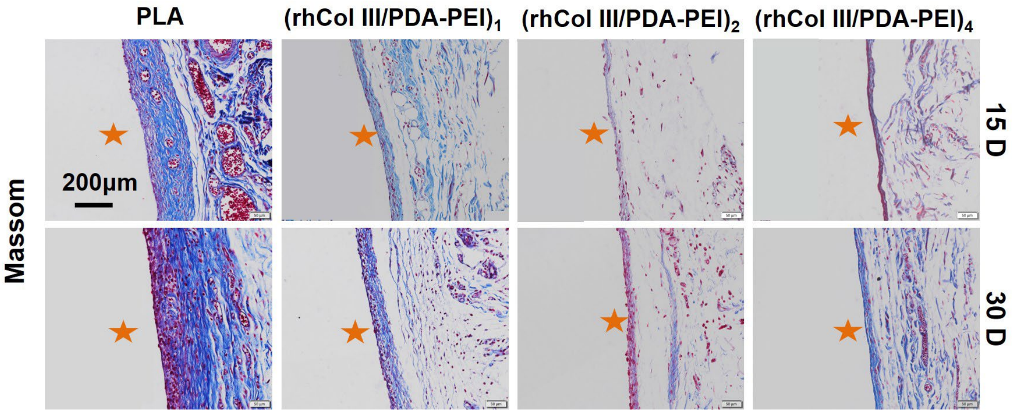

Supplementary Fig. 12 | In vivo $(rhCol\ III/PDA\text{-}PEI)_n$ coatings (n=0.5, 1, 2, and 4) reduce the fiber capsules formation in the rat model. Representative Masson's trichrome staining showing severe fibrosis in the PLA and $(rhCol\ III/PDA\text{-}PEI)_1$ groups compared to the $(rhCol\ III/PDA\text{-}PEI)_n$ groups (n=2 and 4) after subcutaneous implantation for 15 and 30 days. Scale bars, 200 μm. The PLA implants marked by orange stars.

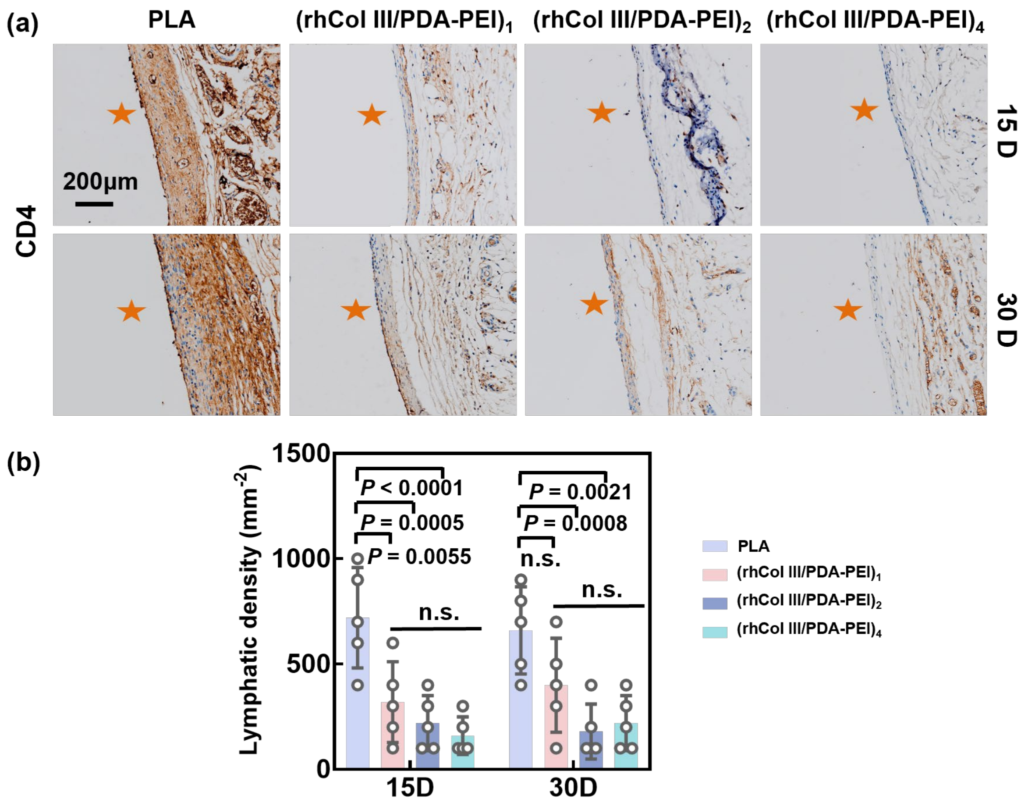

Supplementary Fig. 13 | In vivo (rhCol III/PDA-PEI)$_n$ coatings (n=0.5, 1, 2, and 4) reduce the infiltration of lymphocytes in the rat model. (a) Representative CD4 staining showing more lymphocytes in the PLA and (rhCol III/PDA-PEI)$_1$ groups compared to the (rhCol III/PDA-PEI)$_n$ groups (n=2 and 4) after subcutaneous implantation for 15 and 30 days. Scale bars, 200 μm. The PLA implants marked by orange stars. (b) Corresponding quantification of the numbers of lymphocytes (n= 5 independent samples in independent animals). Two-way ANOVA with Tukey's multiple comparisons was used for the comparisons. The data are presented as the mean ± SD (*P* values <0.05 were considered statistically significant, and n.s. indicated not significant).

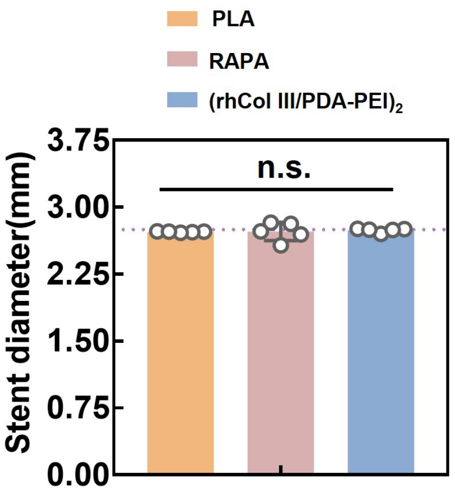

Supplementary Fig. 14 | Quantitative analysis the diameters of PLA-based stents by HE staining. The diameters of PLA-based stents at 3 months post implantation were almost identical and matched well with the reference value (2.75 mm) (n= 5 independent samples in independent animals). One-way ANOVA with Tukey's multiple comparisons was used for the comparisons. The data are presented as the mean ± SD (n.s. indicated not significant).

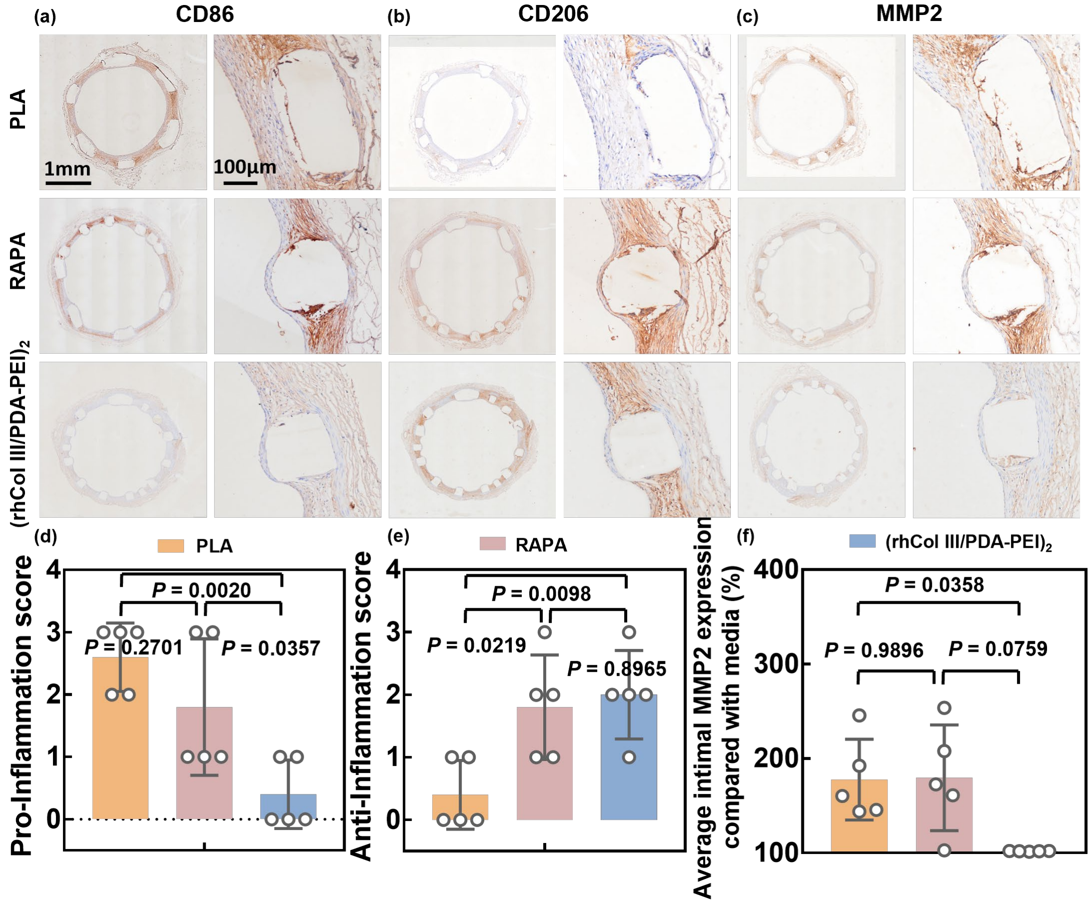

Supplementary Fig. 15 | In vivo (rhCol III/PDA-PEI)$_n$ coatings (n=0.5, 1, 2, and 4)

reduce inflammatory response in the rabbit model. Representative (a) CD86, (b) CD206, and (c) MMP2 immunohistochemical staining of stented abdominal aorta 3 months after stent deployment in the rabbit model. Scale bars, 1 mm and 100 μm. Quantitative results of (d) Pro-Inflammation scores and (e) Anti-Inflammation scores as determined by CD86 and CD206 immunohistochemical images, and (f) MMP2 expression in the intima compared with the media (n= 5 independent samples in independent animals). One-way ANOVA with Tukey's multiple comparisons was used for the comparisons in (d)-(f). The data are presented as the mean ± SD (*P* values <0.05 were considered statistically significant).

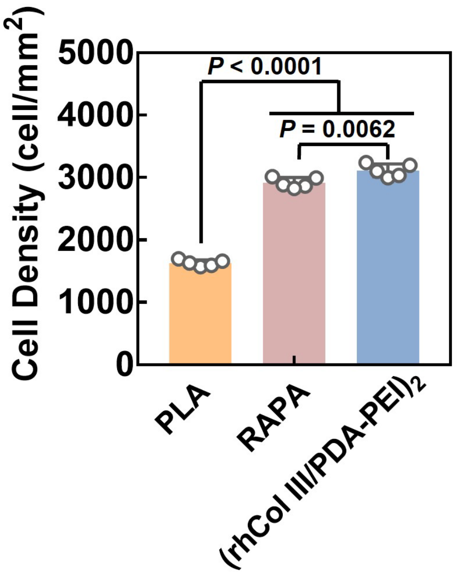

Supplementary Fig. 16 | Cell number of the abdominal aorta implanted with control, RAPA, and (rhCol III /PDA-PEI)$_2$, respectively. Cell density on the surface of the PLA and RAPA stents was lower than that of the (rhCol III/PDA-PEI)$_2$ group. One-way ANOVA with Tukey's multiple comparisons was used for the comparisons (n= 5 independent samples in independent animals). The data are presented as the mean ± SD (*P* values <0.05 were considered statistically significant).

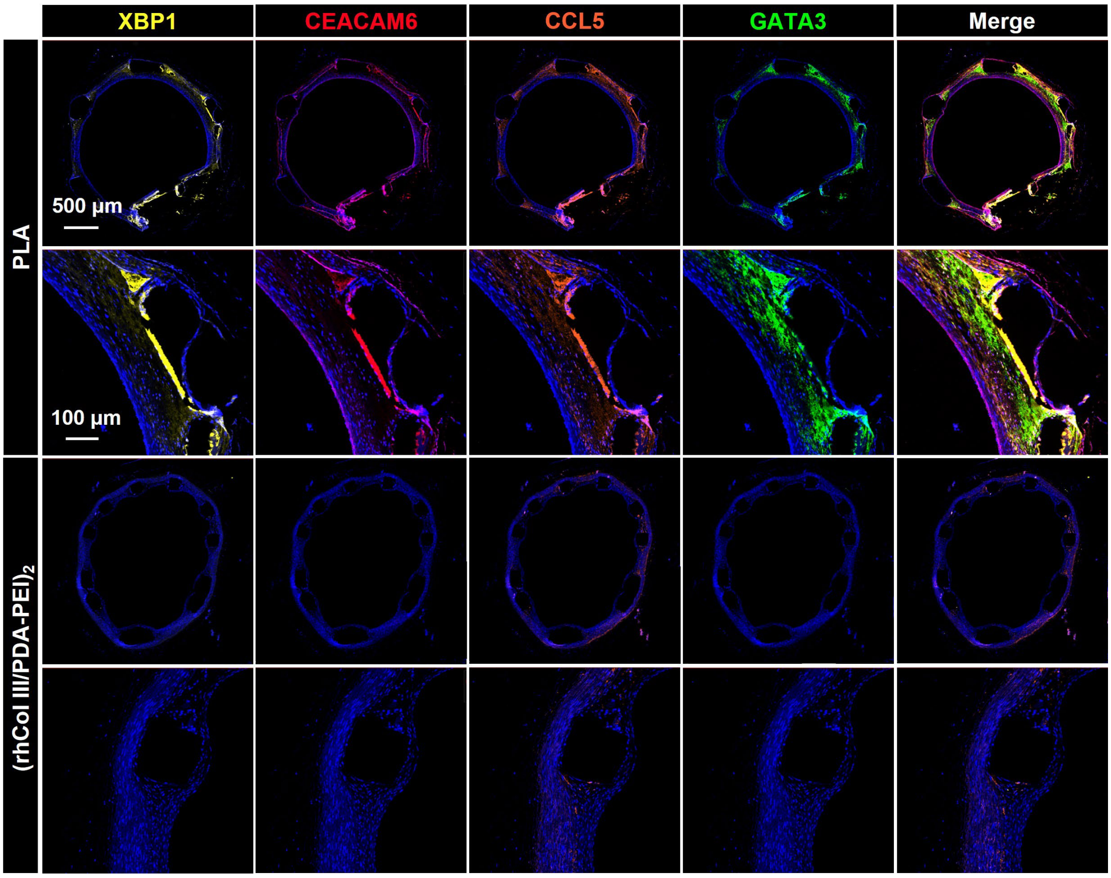

Supplementary Fig. 17 | Representative immunofluorescence staining of the stented abdominal aorta in the rabbit model surface for XBP1 (yellow), CEACAM6 (red), CCL5 (orange), and GATA3 (green), observed by PerkinElmer Vectra Polaris™. High expression of XBP1, GATA3, CEACAM6 and CCL5 were observed in the PLA group with severe intimal hyperplasia, whereas such expressions were significantly inhibited in the (rhCol III/PDA-PEI)$_2$ group with suppressed neointima hyperplasia. Scale bars, 500 μm and 100 μm.

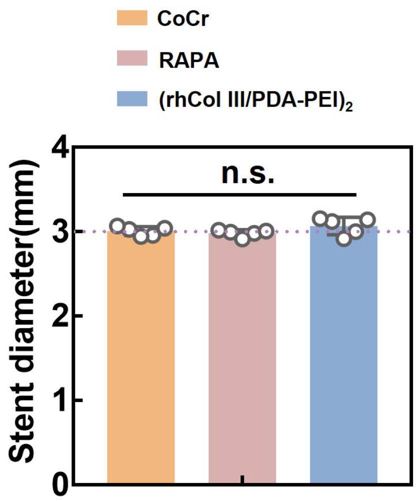

Supplementary Fig. 18 | Diameters of CoCr-based stents. Quantitative analysis determined by HE staining showing the diameters of CoCr-based stents at 3 months post implantation were almost identical and matched well with the reference value (2.75 mm) (n=3 independent samples in independent animals). One-way ANOVA with Tukey's multiple comparisons was used for the comparisons. The data are presented as the mean ± SD (n.s. indicated not significant).

Supplementary Table 1. Abbreviations and full names of genes labeled in the volcano plot of HUVECs

Abbreviation	Full name
DAPK3	Death-associated protein kinase 3
PIK3R1	Phosphoinositide-3-kinase regulatory
XBP1	X-box binding protein 1
CXCL8	C-X-C motif chemokine ligand 8
GATA3	GATA binding protein 3
CEACAM6	CEA cell adhesion molecule 6
CCL5	C-C chemokine ligand 5
NFE2L2	Nuclear factor, erythroid 2 like 2
JADE1	Jade family PHD finger 1
DUSP6	Dual specificity phosphatase 6
LCN2	Lipocalin-2
PEX2	Phosphatidylinositol-3,4,5-trisphosphate dependent Rac exchange
CSF2	colony stimulating factor 2
IL11	Adipogenesis inhibitory factor

ATF3	Activating transcription factor 3
CXCL3	C-X-C motif chemokine ligand 3
CXCL2	C-X-C motif chemokine ligand 8
SDC1	Syndecan-1
AVL9	AVL9 homolog (S. cerevisiase)

Supplementary Table 2. Abbreviations and full names of genes labeled in the volcano plot of HUASMCs

Abbreviation	Full name
MMP7	Matrix metalloproteinase 7
NTF3	Neurotrophic factor-3
CHI3L1	Chitinase 3-like protein 1
TMEM88	Transmembrane protein 88
MAPT	microtubule-associated protein tau gene
MMP9	Matrix metalloproteinase 7
PIR	Private Information Retrieval
FOXN3-AS1	FOXN3 Antisense RNA 1
CCL3	C–C motif chemokine ligand 3
HES6	Basic helix-loop-helix gene Hes6
TBX2-AS1	Gene - TBX2 Antisense RNA 1
PI3	Peptidase Inhibitor 3
IGFBP2	Insulin Like Growth Factor Binding
MMP11	Matrix Metallopeptidase 11
AQP9	Aquaporin 9
MMP2	Matrix Metallopeptidase 2
MGP	Matrix Gla Protein
VIM	Vimentin
LRRC26	Leucine Rich Repeat Containing 26
ACTR2	Actin Related Protein 2
DUSP15	Dual Specificity Phosphatase 15
EGFR	Epidermal Growth Factor Receptor

Supplementary Table 3. Amino acid sequences of the peptides derived from human type III collagen.

ID	Sequence	Purity	Notes
C3P1	Ac-GETGAPGLKGENGLPGENGAPGPMGPRGAP-NH_2	>95 %	1
C3P2	Ac-GQPGPPGPPGTAGFPGSPGAKGEVGPAGSP-NH_2	>95 %	1
C3PO1	Ac-GPAGPOGPOGPOGTSGHOGSOGSOGYQGPO-NH_2	>95 %	2
C3PO2	Ac-GPOGVAGPOGGSGPAGPOGPQGVKGERGSO-NH_2	>95 %	2
T16WTp	Ac-GERGAPGFRGPAGPNGIPGEKGPAGERGAP-NH_2	>95 %	3
T16Op	Ac-GERGAOGFRGPAGPNGIOGEKGPAGERGAO-NH_2	>95 %	4

Note: (Letters in the "Sequence" list are abbreviations for amino acids).

1- Foreseen unfavorite cell or platelet adhesion owing to the absence of GER, GEK sequences or hydroxyproline (O).
2- Foreseen induces platelet adhesion owing to the introduction of hydroxyproline (O).
3- Foreseen favors cell adhesion but not platelet adhesion owing to the introduction of GER and GEK, and the absence of hydroxyproline (O).
4- Foreseen favors both cell and platelet adhesion owing to the introduction of GER, GEK sequences and hydroxyproline (O).

Production of recombinant humanized collagen type III

A total of six peptides derived from human type III collagen (1466 amino acids) associated with platelet and vascular cell adhesion were screened (Supplementary Table S3). Of those, the peptides C3P1 and C3P2 without both GER or GEK sequences and hydroxyproline (O) exhibited no affinity for cell or platelet adhesion. The presence of GPO sequences resulted in peptides C3PO1 and C3PO2 favorable for platelet adhesion. T16WTp, a peptide derived from the Gly483-Pro512 sequence, exhibited a high affinity for cell adhesion but not platelets, which is probably attributed to the retention of highly adhesive fragments (Gly-Glu-Arg (GER) and Gly-Glu-Lys (GEK)) and the bypassing of the hydroxyproline (O) sequence that may induce platelet adhesion and activation. In contrast, T16Op was designed as a mutation of T16WTp by introducing hydroxyproline (O) in contrast to T16WTp by activating more platelets. As a conclusion, T16WTp (sequence: Ac-GERGAPGFRGPAGPNGIPGEKGPAGERGAP-NH_2) was

selected for the meticulous tailoring of the recombinant humanized collagen type III (rhCol III, average Mw ~ 45 KD) with a stable triple-helix conformation based on the requirements for cardiovascular implant devices, which was acquired by tandemly repeating 16 T16WTp with the use of advanced technologies including peptide synthesis and genetic engineering. Subsequently, this protein was expressed in *Escherichia coli (E. coli)* and purified to be endotoxin-free under GMP conditions. The detailed production formulation of Recombinant Humanized Collagen Type III was described in Supplementary Information.

Supplementary Table 4. Primers related to HUVECs growth behavior

Primer name	Orientation	Primer sequence（5'-3'）	Product length
H-TMED2	FORWARD	TCACCAGAACAAGCTAGAAGAA	142
	REVERSE	CCAAAGGACCACTCTGCTGT	
H-DAPK3	FORWARD	GAGAACAAGACGGACGTGGT	147
	REVERSE	AGAGTGCAGGTAGTGAACGC	
H-PIP4K2C	FORWARD	TTTCGTGCAGCAGAAGGTGA	156
	REVERSE	ACCTTGATCTTGGAGCTGGC	
H-PIK3R1	FORWARD	GCTGAAAACCTGTTGCGAGG	108
	REVERSE	ATGCTTTACTTCGCCGTCCA	
H-CXCL8	FORWARD	CTCCAAACCTTTCCACCCCA	127
	REVERSE	TTCCTTGGGGTCCAGACAGA	
H-CEACAM6	FORWARD	TCAAAGGCCAATTACCGTCCA	248
	REVERSE	GCTGAGAGGACAGGAGCACTT	
H-CCL5	FORWARD	ATGACTCCCGGCTGAACAAG	187
	REVERSE	GCCTCCCAAGCTAGGACAAG	
H-NFE2L2	FORWARD	TTCAGCCAGCCCAGCACAT	120
	REVERSE	TCTGCGCCAAAAGCTGCAT	
H-DUSP6	FORWARD	TCGGACATCGAGTCTGACCT	193
	REVERSE	TGGGGGTGACGTTCAAGATG	
H-LCN2	FORWARD	GTTACCTCGTCCGAGTGGTG	126
	REVERSE	CCGAAGTCAGCTCCTTGGTT	
H-PEX2	FORWARD	GGGAACTTCTCTGGCATGGT	177
	REVERSE	TGGGCCACTCTCCACATAGA	
H-HBEGF	FORWARD	CCTCCCAGTGGAAAATCGCT	110
	REVERSE	ACATGAGAAGCCCCACGATG	
H-CXCL3	FORWARD	TGAATGTAAGGTCCCCCGGA	106
	REVERSE	TTCTGAACCATGGGGGATGC	
H-CXCL2	FORWARD	GACCCTGCAGGGAATTCACCTC	141
	REVERSE	TTAACCATGGGCGATGCGGG	

H-XBP1	FORWARD	CGACGGGACCCCTAAAGTTC	237
	REVERSE	CGATCTCTGGCAGTCTGAGC	
H-SDC1	FORWARD	CTCTGGCTCTGGCTGTGC	212
	REVERSE	GTGGGAATAGCCGTCAGGAG	
H-GATA3	FORWARD	ATGCAAGTCCAGGCCCAAG	221
	REVERSE	TCTGACAGTTCGCACAGGAC	
H-SCHIP1	FORWARD	CGTGTTACAACGTGGGACTG	146
	REVERSE	CAAGGGGTCACTTTGGTGGA	
H-β-Actin	FORWARD	AATCTGGCACCACACCTTCTACAA	172
	REVERSE	GGATAGCACAGCCTGGATAGCAA	

Supplementary Table 5. Primers related to macrophage polarization

Primer name	Orientation	Primer sequence（5'-3')	Product length
CD86	FORWARD	CGTTACTCTCCTGCCATCCTTC	212
	REVERSE	CATTTGTGGTGGGAGAAACTGT	
CD206	FORWARD	GAGGATATGAAGCCATGTACTCCT T	199
	REVERSE	TCGCTTCCCTCAAAGTGCAAT	
Arginase-1	FORWARD	CTTGCGAGACGTAGACC	102
	REVERSE	ATCACCTTGCCAATCCC	
IL-6	FORWARD	GACAAAGCCAGAGTCCTTCAGA	76
	REVERSE	TGTGACTCCAGCTTATCTCTTGG	
IL-1β	FORWARD	TGCCACCTTTTGACAGTGATG	220
	REVERSE	AAGGTCCACGGGAAAGACAC	
TNF-α	FORWARD	TCAAAATTCGAGTGACAAGCCTG	245
	REVERSE	GGTATGAGATAGCAAATCGGCTG	
iNOS	FORWARD	CAAGGCCACATCGGATTTCA	174
	REVERSE	TCTATTTTTGCCTCTTTAAAGGAGC	
IL-10	FORWARD	CAACATACTGCTAACCGACTC	77
	REVERSE	GGATCATTTCCGATAAGG	
TGF-β	FORWARD	ACGTGGAAATCAACGGGATCAG	270
	REVERSE	GACAGAAGTTGGCATGGTAGCC	
β-Actin	FORWARD	GTGCTATGTTGCTCTAGACTTCG	174
	REVERSE	ATGCCACAGGATTCCATACC	
