# Supplementary material for: A drug-free cardiovascular stent functionalized with tailored collagen supports in-situ healing of vascular tissues
Source: Nat Commun. 2024 Jan 25;15:735. doi: 10.1038/s41467-024-44902-2 (PMC10810808; doi:10.1038/s41467-024-44902-2)
Supplement: Supplementary file 3 — Reporting Summary [file 41467_2024_44902_MOESM3_ESM.pdf]

nature portfolio

Corresponding author(s): NCOMMS-23-28838-T

Last updated by author(s): Dec 12, 2023

Reporting Summary

Nature Portfolio wishes to improve the reproducibility of the work that we publish. This form provides structure for consistency and transparency in reporting. For further information on Nature Portfolio policies, see our Editorial Policies and the Editorial Policy Checklist.

Statistics

For all statistical analyses, confirm that the following items are present in the figure legend, table legend, main text, or Methods section.

n/a	Confirmed	
☐	☒	The exact sample size (*n*) for each experimental group/condition, given as a discrete number and unit of measurement
☐	☒	A statement on whether measurements were taken from distinct samples or whether the same sample was measured repeatedly
☐	☒	The statistical test(s) used AND whether they are one- or two-sided<br>*Only common tests should be described solely by name; describe more complex techniques in the Methods section.*
☒	☐	A description of all covariates tested
☒	☐	A description of any assumptions or corrections, such as tests of normality and adjustment for multiple comparisons
☐	☒	A full description of the statistical parameters including central tendency (e.g. means) or other basic estimates (e.g. regression coefficient) AND variation (e.g. standard deviation) or associated estimates of uncertainty (e.g. confidence intervals)
☐	☒	For null hypothesis testing, the test statistic (e.g. *F*, *t*, *r*) with confidence intervals, effect sizes, degrees of freedom and *P* value noted<br>*Give P values as exact values whenever suitable.*
☒	☐	For Bayesian analysis, information on the choice of priors and Markov chain Monte Carlo settings
☒	☐	For hierarchical and complex designs, identification of the appropriate level for tests and full reporting of outcomes
☒	☐	Estimates of effect sizes (e.g. Cohen's *d*, Pearson's *r*), indicating how they were calculated

Our web collection on statistics for biologists contains articles on many of the points above.

Software and code

Policy information about availability of computer code

Data collection	Data in Figs. 2a, 2d, 2f 4a, 5a, 6j,Supplementary Fig. 1, Supplementary Fig. 7, Supplementary Fig. 9a, and Supplementary Fig. 10 were observed by confocal laser scanning microscope (CLSM) (Leica SP5, Germany).<br>Data in Fig. 2b was observed by spectroscopic ellipsometer (M-2000 V, J.A. Woollam, USA) measurement.<br>Data in Fig. c observed by attention theta (Biolin Scientific, Sweden).<br>Data in Figs. 2e, 3b, 3f, 8b, and Supplementary Figs. 2-5 were observed by scanning electron microscopy (SEM, FEI Nova NanoSEM 450).<br>Data in Figs. 2g-2m were observed by X-ray photoelectron spectroscopy (XPS, Thermo Scientific ESCALAB 250Xi, USA).<br>Data in Fig. 2n was observed by QSense Analyzer instrument (Biolin Scientific AB, Sweden) .<br>Data in Fig. 2o was observed by surPASS 3 (Anton Paar, Austria) instrument.<br>Data in Figs. 4f-4i and 5d-5f were collected using the TruSeq SR Cluster Kit v3-cBot-HS (Illumina, Inc., USA).<br>Data in Figs. 4j, 5g-5i, and 6d were collected using CFX96 instrument (Bio-Rad).<br>Data in Figs. 4k, 6a, 7a, 7c, 8c-8e, 8n, Supplementary Fig. 12, Supplementary Fig. 13a, and Supplementary Fig. 15a were observed by a whole slide scanner (VS200, Olympus).<br>Data in Supplementary Fig. 17 was collected using PerkinElmer Vectra Polaris™ (Akoya, Vectra Polaris).<br>All softwares used in this study have been described in detail in methods part.
Data analysis	XPS dates were analyzed with the curve-fitting program (CasaXPS, Version 2.3.17PR1.1). The analysis of QCM-D date was conducted with Dfind Smartfit modeling in QSense Dfind software (QSoft401). Cell counts were calculated by ImageJ software (1.52a, NIH, USA). RT-PCR date was quantified using the 2-ΔΔCt formula with Line Gene 9600 Plus Software (FQD-96A, Bio-Rad, Japan). The RNA sequencing was conducted on an Illumina HiSeq 4000 by using the TruSeq SR Cluster Kit v3-cBot-HS (Illumina, Inc., USA). Solexa pipeline version 1.8 (off-line base caller software, version 1.8) was adopted to perform image processing and base recognition; Genome version used in reads mapping is GenCode

GRCh37. Differentially expressed mRNAs (DEmRNAs) were determined by the Ballgown package . Volcano maps were generated using the ggscatter R package (1.18.0). Hierarchical bidirectional clustering analysis was performed by using the heatmap R package. GO analyses were subjected to statistical calculations and graphing using the topGO software package in the R environment, and pathway analyses were calculated. Histomorphometric, immunohistochemical, and immunofluorescent staining results were analyzed
by ImageJ software (1.52a, NIH, USA). Optical coherence tomography (OCT) images were obtained by RadiAnt DICOM Viewer (2020.2.3).
Other date were analyzed by GraphPad Prism (7. 04).
All softwares used in this study have been described in detail in methods part.

For manuscripts utilizing custom algorithms or software that are central to the research but not yet described in published literature, software must be made available to editors and reviewers. We strongly encourage code deposition in a community repository (e.g. GitHub). See the Nature Portfolio guidelines for submitting code & software for further information.

Data

Policy information about availability of data

All manuscripts must include a data availability statement. This statement should provide the following information, where applicable:

- Accession codes, unique identifiers, or web links for publicly available datasets
- A description of any restrictions on data availability
- For clinical datasets or third party data, please ensure that the statement adheres to our policy

The main data supporting the results of this study are available within the paper and its Supplementary Information. All other additional data generated in this study are available for research purposes from the corresponding authors upon reasonable request. The raw HUVECs and HUASMCs transcriptome data have been deposited into the Genome Sequence Archive (GSA: HRA005037 for HUVECs; GSA: HRA005839 for HUASMCs) and are publicly accessible at https://ngdc.cncb.ac.cn/gsa. Furthermore, there is an additional list of figures that have associated source data: Fig. 2b, Fig. 2c, Fig. 2g, Fig. 2h, Fig. 2i, Fig. 2j, Fig. 2k, Fig. 2l, Fig. 2m, Fig. 2n, Fig. 2o; Fig. 3c, Fig. 3d, Fig. 3h, Fig. 3i; Fig. 4b, Fig. 4c, Fig. 4d, Fig. 4e, Fig. 4f, Fig. 4g, Fig. 4h, Fig. 4i, Fig. 4j; Fig. 5b, Fig. 5c, Fig. 5d, Fig. 5e, Fig. 5f, Fig. 5g, Fig. 5h, Fig. 5i; Fig. 6b, Fig. 6c, Fig. 6d; Fig. 7b, Fig. 7d, Fig. 7e; Fig. 8f, Fig. 8g; Fig. 8h, Fig. 8i, Fig. 8k, Fig. 8l, Fig. 8o, Fig. 8p, and Supplementary Figs. 6; 8; 9b, 9c; 13b; 14; 15d-15f; 16; 17; 18.
A data availability statement has been provided in manuscript.

Research involving human participants, their data, or biological material

Policy information about studies with human participants or human data. See also policy information about sex, gender (identity/presentation), and sexual orientation and race, ethnicity and racism.

Reporting on sex and gender	NA
Reporting on race, ethnicity, or other socially relevant groupings	NA
Population characteristics	NA
Recruitment	NA
Ethics oversight	NA

Note that full information on the approval of the study protocol must also be provided in the manuscript.

Field-specific reporting

Please select the one below that is the best fit for your research. If you are not sure, read the appropriate sections before making your selection.

☒ Life sciences ☐ Behavioural & social sciences ☐ Ecological, evolutionary & environmental sciences

For a reference copy of the document with all sections, see nature.com/documents/nr-reporting-summary-flat.pdf

Life sciences study design

All studies must disclose on these points even when the disclosure is negative.

Sample size	For physicochemical experiments related to the characterization of materials and in vitro cell experiments, n=3 was chosen as the minimal replicate numbers based on previous studies including our group that reported similarly characterized experiments (Nature communications. 2019,10(1):3491; Science advances.2022,8(9):eabm3378; Nature biomedical engineering. 2021,5(10): 1174-1188).<br>For experiments involving in vitro hemocompatibility assay, in vivo subcutaneous implantation in rat model experiments, and in vivo stent implantation in rabbit model experiments, sample size (n=5) was chosen based on previous studies of similar characterized experiments (Nature biomedical engineering. 2021,5(10): 1174-1188).<br>For in vivo stent implantation in pig model experiments, sample size (n=3) was chosen based onbased on experience and existing literature on specific animal models (Cell. 2018,173(4): 989-1002).<br>In conclusion, all sample sizes in this study are consistent with previously published works.
Data exclusions	No data were excluded from the analyses in this study.

April 2023

Replication	All experiments were performed in n independent replicates. The n number is specified in the text and or the figure legends
Randomization	Male Sprague Dawley rats, New Zealand white adult rabbits, and Miniature pigs used in this study were randomly selected for control or experimental groups and kept in separate cages throughout the experiment. Rest other in vitro and ex vivo experiments each group was randomly selected.
Blinding	No formal blinding was used in this study. Because all experiments were performed based on standardized protocols and blinding has no effect on the experiment results. Unbiased experimental procedure and date analysis were carried out as far as possible.

Reporting for specific materials, systems and methods

We require information from authors about some types of materials, experimental systems and methods used in many studies. Here, indicate whether each material, system or method listed is relevant to your study. If you are not sure if a list item applies to your research, read the appropriate section before selecting a response.

Materials & experimental systems

n/a	Involved in the study	
☐	☒	Antibodies
☐	☒	Eukaryotic cell lines
☒	☐	Palaeontology and archaeology
☐	☒	Animals and other organisms
☒	☐	Clinical data
☒	☐	Dual use research of concern
☒	☐	Plants

Methods

n/a	Involved in the study	
☒	☐	ChIP-seq
☒	☐	Flow cytometry
☒	☐	MRI-based neuroimaging

Antibodies

Antibodies used	Primary antibodies used in this study included mouse monoclonal XBP1 (Cat. No.: sc-8015, Lot. No.: B0523, Clone: F-4, Santa Cruz Biotechnology, USA, 1:50), mouse polyclonal CCL5 (Cat. No.: sc-365826, Lot. No.: H1022, Clone: A-4, Santa Cruz Biotechnology, USA, 1:50), mouse monoclonal CEACAM6 (Cat. No.: sc-59899, Lot. No.: A0816, Clone: 9A6, Santa Cruz Biotechnology, USA, 1:50), rabbit monoclonal GATA3 (Cat. No.: ab199428, Lot. No.: 1010814-13, Clone: EPR16651, Abcam, USA, 1:500), rabbit polyclonal F4/80 (Cat. No.: 29414-1-AP, Lot. No.: 00098190, Proteintech, China, 1:100), rabbit monoclonal CD68 (Cat. No.: ab283654, Lot. No.: GR3405514-1, Clone: EPR23917-164, Abcam, USA, 1:100), rabbit polyclonal CD86 (Cat. No.: bs-1035R, Lot. No.: BC03035003, Biosynthesis Biotechnology co., ltd, USA, 1:200), rabbit monoclonal CD206 (Cat. No.: 24595, Lot. No.: 3, Clone: E6T5J, Cell Signaling Technology, USA, 1:200), mouse monoclonal α-SMA (Cat. No.: ab7817, Lot. No.: GR3425194-7, Clone: 1A4, Abcam, USA, 1:200), and rabbit monoclonal MMP2 (Cat. No.: 10373-2-AP, Lot. No.: 00124042, Clone: SB13a, Proteintech, USA, 1:200), mouse monoclonal CD31 (Cat. No.: ab9498, Lot. No.: GR3384066-4, Clone: JC/70A, Abcam, USA, 1:200), and rabbit polyclonal eNOS (Cat. No.: ab5589, Lot. No.: 1035207-4, Abcam, USA, 1:100). Secondary antibodies used in this study included Alex Fluor 488 goat anti-rabbit IgG (Cat. No.: A-11008, Lot. No.: BC03035003, Lot. No.: A11034, Invitrogen, USA, 1:500), Alex Fluor 488 goat anti-mouse IgG (Cat. No.: A-11001, Lot. No.: BC03035003, 2465113, Invitrogen, USA, 1:500), and Alexa Fluor647 donkey anti-rabbit IgG (Cat. No.: A-31573,Lot. No.: 2420695, Invitrogen, USA, 1:500).
Validation	All primary antibodies used in this study are commercially available and validated by the manufacturers<br>XBP1:https://www.scbt.com/p/xbp-1-antibody-f-4?requestFrom=search<br>CCL5:https://www.scbt.com/p/rantes-antibody-a-4?requestFrom=search<br>CEACAM6:https://www.scbt.com/p/ceacam6-antibody-9a6?requestFrom=search<br>GATA3:https://www.abcam.cn/products/primary-antibodies/gata3-antibody-epr16651-chip-grade-ab199428.html<br>F4/80:https://www.biomart.cn/infosupply/102845897.htm#102846102<br>CD68:https://www.abcam.cn/products/primary-antibodies/cd68-antibody-epr23917-164-ab283654.html<br>CD86:http://bioss.com.cn/prolook_03.asp?id=AF08169606000209&pro37=1<br>CD206:https://www.cellsignal.cn/products/primary-antibodies/cd206-mrc1-e6t5j-xp-rabbit-mab/24595<br>α-SMA:https://www.abcam.cn/products/primary-antibodies/alpha-smooth-muscle-actin-antibody-1a4-ab7817.html<br>MMP2:https://www.ptgcn.com/products/MMP2-Antibody-10373-2-AP.htm<br>CD31:https://www.abcam.cn/products/primary-antibodies/cd31-antibody-jc70a-ab9498.html<br>eNOS:https://www.abcam.cn/products/primary-antibodies/enos-antibody-ab5589.html<br>Alex Fluor 488 goat anti-rabbit IgG:https://www.thermofisher.cn/cn/zh/antibody/product/Goat-anti-Rabbit-IgG-H-L-Cross-Adsorbed-Secondary-Antibody-Polyclonal/A-11008<br>Alex Fluor 488 goat anti-mouse IgG:https://www.thermofisher.cn/cn/zh/antibody/product/Goat-anti-Mouse-IgG-H-L-Cross-Adsorbed-Secondary-Antibody-Polyclonal/A-11001<br>Alexa Fluor647 donkey anti-rabbit IgG:https://www.thermofisher.cn/cn/zh/antibody/product/Donkey-anti-Rabbit-IgG-H-L-Highly-Cross-Adsorbed-Secondary-Antibody-Polyclonal/A-31573

Eukaryotic cell lines

Policy information about cell lines and Sex and Gender in Research

Cell line source(s)	Primary human umbilical vein endothelial cells (HUVECs, Cat. No.: STCC12103) and Primary human umbilical artery smooth muscle cells (HUASMCs, Cat. No.: HTX2180) were both purchased from Service-bio Technology Co. Ltd. (China), and Primary mouse bone marrow-derived macrophages (MBMMCs, Cat. No.: CP-M172) were obtained from Procell Life Science&Technology Co., Ltd. (China).
Authentication	Authentication of the cells was provided by Service-bio Technology Co. Ltd. and Procell Life Science&Technology Co., Ltd. and validated by the expression of surface markers.
Mycoplasma contamination	I confirm that all cell lines tested negative for mycoplasma contamination.
Commonly misidentified lines (See ICLAC register)	No commonly misidentified lines used in the study.

Animals and other research organisms

Policy information about studies involving animals; ARRIVE guidelines recommended for reporting animal research, and Sex and Gender in Research

Laboratory animals	Male Sprague Dawley rats (SD rats, ~ 250-300 g, ~10 weeks old) and New Zealand white adult rabbits (~2.5-3 kg, ~3 months old) were purchased from Chengdu Dossy Experimental Animals Co., Ltd. Miniature pigs (~ 35 kg, ~6 months old) were provided by Amsinomed Medical Co., Ltd.
Wild animals	No wild animals in this study
Reporting on sex	The analysis of sex-dependent differences was not pursued in this study. The Laboratory animals were provided by Chengdu Dossy Experimental Animals Co., Ltd. and Amsinomed Medical Co., Ltd.
Field-collected samples	The study did not involve samples collected from that field.
Ethics oversight	All animal experiments were kept to a strict protocol approved by the medical ethics committee of Sichuan Provincial and conducted with the guidelines for the care and use of laboratory animals of Sichuan University (No. KS2020394).

Note that full information on the approval of the study protocol must also be provided in the manuscript.
